# New vitamin B_12_ derivatives activates sGC

**DOI:** 10.1186/1471-2210-11-S1-P32

**Published:** 2011-08-01

**Authors:** Dorota Gryko, Emil Martin

**Affiliations:** 1 Institute of Organic Chemistry Polish Academy of Sciences, Warsaw, Poland; 2 University of Texas, Graduate School o Biomedical Science, Houston, USA

## Background

Protoporphyrin IX (PPIX) was shown to strongly activate *in vitro* the soluble guanylate cyclase enzyme (sGC), which makes it an interesting drug candidate for treatment of hypertension [1,2]. In order to overcome the problem of PPIX poor bioavailability (especially in the case of oral administration), we decided to exploit the specific uptake pathway of vitamin B_12_, which was frequently used for delivering biologically active substances from the digestive system into the body cells. To this end, we embarked on the synthesis of a series of hybrid molecules, containing PPIX and vitamin B_12_ moieties, linked *via* chains of different length and chemical character [3].

## Methods and results

Our synthetic approach is based on the synthesis of linking molecules containing a primary -NH_2_ group and -N_3_ or alkyne group at the other end. Amine functionality allows us to connect these linkers to vitamin B_12_, and to PPIX, finally, the union of the two parts is possible using Cu-catalyzed azide alkyne cycloaddition (the “click reaction”) [4].
